# P-1373. Breathless: An Outbreak of Silico-Tuberculosis Among Engineered Stone Countertop Fabrication Workers in Los Angeles County

**DOI:** 10.1093/ofid/ofaf695.1560

**Published:** 2026-01-11

**Authors:** David L Fuller, Paul Holden

**Affiliations:** Los Angeles County Department of Public Health, Los Angeles, CA; Los Angeles County, Santa Barbara, California

## Abstract

**Background:**

Silicosis is a known risk factor for the development of tuberculosis (TB) disease, occurring commonly among miners. “Silico-tuberculosis” (silico-TB) describes individuals affected by both silicosis and TB. Here we characterize an outbreak of silico-TB among stone countertop fabrication workers in Los Angeles County (LAC).

**Methods:**

In 2024, we were alerted to new TB incidents with genotypic and/or epidemiologic linkage (epi-link) to a cluster first identified in workers at a stone countertop fabrication facility in 2015. An investigation was conducted using data from LAC Department of Public Health (DPH) electronic health records, contact investigation (CI) and TB registry databases, and Centers for Disease Control TB Genotyping Information Management System (GIMS). Data elements, including CI outcomes and genotypes, were extracted. TB was diagnosed by sputum culture, and confirmed/suspected silicosis was diagnosed based on occupational history, chest imaging, and/or pathology.

**Results:**

From 2015-2025, six cases of genetically similar silico-TB were diagnosed. Common features in this cohort included sputum acid-fast bacilli (AFB) smear positivity, cavitary chest imaging, and history of employment at stone countertop fabrication facility. Five patients completed or are undergoing TB treatment. One death attributed to TB occurred during treatment. Case A was diagnosed with pulmonary TB in 2015. Retrospective review of chest imaging suggests missed diagnosis of silicosis at time of initial presentation. Case B was diagnosed in 2021. No definite epi-link to case A was confirmed as case B died prior to completion of their CI. Two coworkers (C and D) of case A were diagnosed with latent TB infection (LTBI) during case A’s CI, but neither individual completed preventive treatment. Cases C and D were later diagnosed with silico-TB in 2023 and 2024, respectively. Case E was diagnosed in 2024 and identified cases A and D as coworkers. Case F was diagnosed in 2025 and identified cases A and B as coworkers.

**Conclusion:**

An outbreak of silico-TB was identified among stone countertop fabrication workers in LAC. Prioritization of LTBI screening and preventive treatment completed via directly observed therapy for these workers may help prevent future cases of silico-TB.

**Disclosures:**

All Authors: No reported disclosures

